# Lipid-Based Formulation of Baricitinib for the Topical Treatment of Psoriasis

**DOI:** 10.3390/pharmaceutics16101287

**Published:** 2024-09-30

**Authors:** Roya Mohammadi-Meyabadi, Mireia Mallandrich, Negar Beirampour, Núria Garrós, Lupe Carolina Espinoza, Lilian Sosa, Joaquim Suñer-Carbó, María José Rodríguez-Lagunas, María Luisa Garduño-Ramírez, Ana C. Calpena-Campmany

**Affiliations:** 1 Departament de Farmàcia i Tecnologia Farmacèutica, i Fisicoquímica, Facultat de Farmàcia i Ciències de l’Alimentació, Universitat de Barcelona (UB), Av. Joan XXIII, 27-31, 08028 Barcelona, Spain; rmohammo31@alumnes.ub.edu (R.M.-M.); nbeirabe7@alumnes.ub.edu (N.B.); jsuner@ub.edu (J.S.-C.); anacalpena@ub.edu (A.C.C.-C.); 2 Institut de Nanociència i Nanotecnologia, Universitat de Barcelona (UB), Av. Diagonal 645, 08028 Barcelona, Spain; 3 Departamento de Química y Ciencias Exactas, Universidad Técnica Particular de Loja, Loja 1101608, Ecuador; 4 Pharmaceutical Technology Research Group, Faculty of Chemical Sciences and Pharmacy, National Autonomous University of Honduras (UNAH), Tegucigalpa 11101, Honduras; 5 Instituto de Investigaciones en Microbiología (IIM), Universidad Nacional Autónoma de Hondura (UNAH), Tegucigalpa 11101, Honduras; 6 Departament de Bioquímica i Fisiologia, Facultat de Farmàcia i Ciències de l’Alimentació, Universitat de Barcelona (UB), Av. Joan XXIII, 08028 Barcelona, Spain; 7 Nutrition and Food Safety Research Institute (INSA-UB), 08921 Santa Coloma de Gramenet, Spain; 8 Centro de Investigaciones Químicas, Instituto de Investigación en Ciencias Básicas y Aplicadas, Universidad Autónoma del Estado de Morelos, Av. Universidad 1001, Cuernavaca 62209, Mexico

**Keywords:** baricitinib, lipid-based formulations, oily solutions, psoriasis, skin inflammation imiquimod

## Abstract

Background: Baricitinib, commonly used for autoimmune diseases, is typically administered orally, which can lead to systemic adverse effects. A topical formulation could potentially offer localized therapeutic effects while minimizing these side effects. Objectives: This study focuses on developing a lipid-based topical formulation of baricitinib (BCT-OS) for treating psoriasis. Methods: The optimized formulation was then assessed for physical, chemical, and biopharmaceutical characterization. Furthermore, the anti-inflammatory efficacy of the formulation was tested in a model of psoriasis induced by imiquimod in mice, and its tolerance was determined by the evaluation of biomechanical skin properties and an inflammation test model induced by xylol in mice. Results: BCT-OS presented appropriate characteristics for skin administration in terms of pH, rheology, extensibility, and stability. The formulation also demonstrated a notable reduction in skin inflammation in the mouse model, and high tolerability without affecting the skin integrity. Conclusions: BCT-OS shows promise as an alternative treatment for psoriasis, offering localized therapeutic benefits with a potentially improved safety profile compared to systemic administration.

## 1. Introduction

Psoriasis is a chronic inflammatory skin disease with a worldwide prevalence of 1–3%, and is characterized by well-defined erythematous papules and plaques covered with silvery-white scales that can vary in intensity and be distributed in localized areas, including elbows, knees, lumbar region, armpits, groin, scalp, and nails [1]. This disease affects both men and women, but appears earlier in women and those with a family history [2]. Due to the chronicity and severity of the disease, it can negatively impact the quality of life of patients at the mental, physical, and economic level [3]. The pathophysiology of psoriasis involves irregular keratinocyte proliferation and the infiltration of immune cells into both the dermis and epidermis. This process engages the innate and adaptive immune systems, dendritic cells and T cells playing important roles [4,5].

The treatments available for this disease include topical agents such as urea and salicylic acid ointments, corticosteroids alone or in combination with vitamin D analogues, retinoids, and calcineurin inhibitors. Other treatments used in psoriasis are phototherapy and oral or injectable treatments with steroids, retinoids, and biological drugs that target TNF, IL-12, IL-23, and IL-17 [6]. Several oral treatments are currently under development including phosphodiesterase (PDE) 4 inhibitors such as apremilast and roflumilast, oral interleukin (IL)-17 inhibitors such as tildrakizumab and Janus kinase (JAK) inhibitors such as tofacitinib, abrocitinib, peficitinib, and baricitinib (BCT) [7,8,9,10,11].

BCT is a drug that selectively inhibits JAK1/JAK2 tyrosine kinases. These kinases are located at the ends of cytokine receptors on the cell membrane and control the signal transduction of cytokines, including those in the IL-6, IL-10, IL-3, and IL-5 families. Each cytokine receptor is bound to two parallel isomers of JAK that exist as homodimers or heterodimers. Upon cytokine binding to their receptors, JAK undergoes phosphorylation, which in turn leads to the phosphorylation of STAT proteins (Signal transducer and transcription activator) in the cells. These STAT proteins are then transported to the nucleus where they interact directly with the cell’s DNA to regulate gene expression [12]. This drug has been used in the management and treatment of rheumatoid arthritis, atopic dermatitis [13], and in recent years, in combination with other drugs for the treatment of COVID-19 [14]. Papp and coworkers evaluated the efficacy of oral baricitinib in managing psoriasis in a double-blind controlled study. The study demonstrated a significant improvement in the incidence and prevention of moderate to severe psoriasis in patients after 12 weeks of oral baricitinib treatment, resulting in a 75% reduction in the area [10].

**Figure 1:**
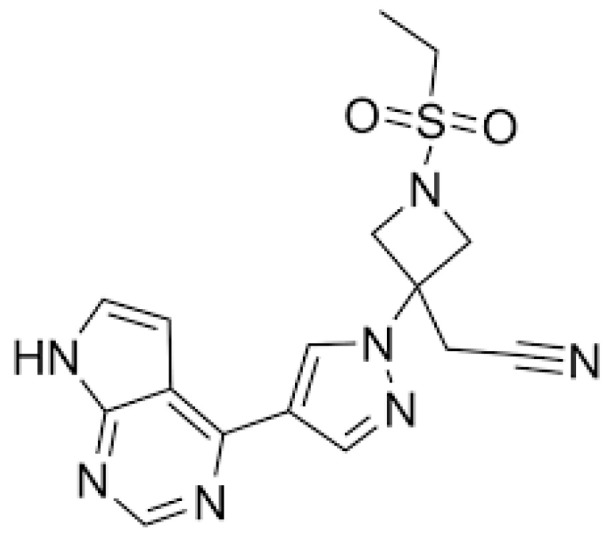
Chemical structure of baricitinib.

BCT is commercially available in the form of tablets for oral administration (Olumiant^®^). The administration of this drug has adverse effects such as immunosuppression, the reactivation of herpes zoster, upper and lower respiratory tract infections, low red and white blood cell counts, significant cardiovascular adverse effects, and an increased risk of malignant neoplasms, and should be used with caution in patients over 65 years of age and diabetics. Before using baricitinib orally, a series of tests such as tuberculosis, HIV, viral hepatitis B or C, and liver function should be performed [15]. Figure 1 displays the chemical structure of BCT.

Administering drugs topically to the skin offers several advantages, including local treatment, minimized systemic side effects, and greater patient acceptance [16]. However, conventional topical formulations often face challenges such as poor solubility, limited skin penetration, and inadequate controlled release [17]. Drug delivery directly to the site of action through the skin can avoid first-pass metabolism and systemic degradation, thereby increasing drug bioavailability. There is growing interest in developing topical treatments using appropriate vehicles to improve adherence in patients with chronic skin diseases such as psoriasis [18].

Lipid topical solutions offer notable advantages for the treatment of dermatological disorders in which the integrity of the skin is compromised since their components can act as moisturizers, humectants, and emollients, generating a protective barrier for the skin [19]. Topical oily solutions consist of a mixture of skin-friendly oily substances that help fill the spaces between intercorneocyte clusters to improve skin hydration, smoothness, softness, and suppleness. They frequently create an occlusive effect by preserving the skin’s moisture through a hydrophobic barrier, which prevents transepidermal water loss. In addition, they are used as vehicles to solubilize water-insoluble drugs ensuring even application, protection, and delivery to deeper layers of the skin. [20].

Based on this background, the objective of this research was to design, develop, and characterize physically, chemically, and biopharmaceutically a lipid-based solution of BCT 5 mg/mL (BCT-OS) as an alternative topical treatment for psoriasis.

## 2. Materials and Methods

### 2.1. Chemicals and Reagents

The bulk powder of baricitinib (BCT) was sourced from Henrikang Biotech Co., Ltd. (Xi’an, China). Transcutol^®^ P (diethylene glycol monoethyl ether), Labrafac^®^ Lipophile WL 1349 (medium chain triglycerides), and Lauroglycol^®^ 90 (propylene glycol monolaurate) were kindly donated by Gattefossé (Saint-Priest, France). Surfadone^®^ LP 100 (N-Octyl-2-Pyrrolidone) was acquired from ISP (Yorkshire, UK), and imiquimod (IMQ) 50 mg/gr cream was obtained from Cantabria Labs (Madrid, Spain). Both distilled and purified water were produced using a Station 9000 purification unit.

### 2.2. Biological Material

Human skin was used to perform the ex vivo permeation studies. The human skin was sourced from abdominoplasty procedures in healthy women. The study protocol received approval from the Bioethics Committee of the Barcelona-SCIAS Hospital (Protocol N°002; dated 17 January 2020). The ex vivo skin was preserved at −20 °C until it was used in the permeation studies.

### 2.3. Analytical Method for Drug Quantification

BCT was determined by an HPLC method coupled to a fluorescence detector (HPLC-FLD). The system consisted of a Waters Alliance 2695 chromatograph (Waters, Milford, MA, USA) and a Jasco FP-1520 fluorescence detector (Jasco Corporation, Tokyo, Japan) at an excitation wavelength (EX) of 310 nm and an emission wavelength (EM) of 390 nm. The mobile phase was composed of 10 mM ammonium formate at pH 7: ACN (75:25 *v*/*v*). The flow rate was set at 1 mL/min under isocratic elution. The stationary phase was a Symmetry C18 column 4.6 × 75 mm, 3.5 μm (Waters Corporation, Cerdanyola del Vallès, Spain) and 10 μL of the samples was injected. A stock solution of 60 μg/mL BCT in Transcutol^®^ P was prepared, then the calibration curve was obtained by sequential dilutions in Transcutol^®^ P–PBS pH 7.4 (1:1, *v*/*v*) within the concentration range of 0.063–1 μg/mL.

### 2.4. Design and Preparation of Lipid-Based Topical Solutions of BCT 5 mg/mL

Different formulations were prepared using Transcutol^®^ P as cosolvent to solubilize the drug. Additionally, different oily vehicles such as Labrafac^®^ Lipophile WL 1349, Lauroglycol^®^ 90, and Surfadone^®^ LP 100 were used in different proportions (Table 1). First, Lauroglycol^®^ and Surfadone^®^ LP 100 were mixed in a ratio (5:2) using a magnetic stirrer for 10 min to ensure proper homogeneity. BCT was weighed in a vial and then the other components were added and sonicated in an ultrasound bath for 30 min to completely solubilize the drug. Finally, the different formulations were equilibrated for 24 h at room temperature. The selection of the optimized formulation was based on the physical and chemical stability evaluated over 30 days of storage at 30 °C and 40 °C.

### 2.5. Characterization of BCT-OS

#### 2.5.1. pH

The pH of the lipid-based formulations was determined using a Crison micropH 2000 digital pH meter (Crison Instruments SA, Alella, Spain). To measure pH, 1 mL of formulation was combined with 30 mL of sterilized water and subjected to an ultrasound bath for 3 min. The electrode was directly immersed in the sample contained in a glass vial. Measurements were performed at room temperature 24 h after the formulations were prepared [21] and the results were reported as mean ± SD (*n* = 3).

#### 2.5.2. Drug Content

The BCT content in the formulation was quantified using a spectrophotometer PerkinElmer UV/Vis (Shelton, CT, USA). BCT-OS was diluted in Transcutol^®^ P to obtain the theoretical concentration within the standard solutions of the calibration curve (50–1.562 µg/mL). The calibration curve was prepared using Transcutol^®^ P; a blank of the formulation was diluted following the same process of BCT-OS to use as a blank solution, and absorption measurements were performed using a quartz cuvette at 310 nm [22].

#### 2.5.3. Rheological Properties

The rheological behaviour of the formulation was evaluated at 25 °C using a rotational rheometer Haake Rheostress^®^ 1 (Thermo Fisher Scientific, Karlsruhe, Germany) equipped with a cone plate setup with a fixed lower plate and a mobile upper cone Haake C60/2° Ti (60 mm diameter, 2° angle). The rheometer was connected to a computer running Haake Rheowin^®^ Data Manager v. 4.91 software (Thermo Electron Corporation, Karlsruhe, Germany) to carry out the test and Haake Rheowin^®^ Data Manager v. 4.91 software (Thermo Electron Corporation, Karlsruhe, Germany) to analyze the obtained data. The shear rate ramp program (viscosity curves and flow curves) involved gradually increasing the shear from 0 to 100 s^−1^ over 3 min, maintaining a constant shear rate of 100 s^−1^, for 1 min, and then gradually decreasing it back to 0 s^−1^ over 3 min. During this process, the shear stress (τ) was measured as a function of the shear rate (γ), providing the viscosity curves (η = f(γ)) and flow curves (τ = f(γ)).

#### 2.5.4. Extensibility

The extensibility assay was conducted to evaluate the spreading behavior of the topical product under varying degrees of pressure. This assay helps understanding the product’s performance when different forces are applied. The extensibility was evaluated in triplicate using an extensometer (custom-designed and built in-house). The extensiometer consists of a base with a mobile center that moves up and down with a lever. When the center is down, the base forms a hole where the formulation is allocated. A transparent and graded upper plaque is then placed on the base, and the lever is shifted to raise the center base compressing the formulation and causing it to spread. The surface area of the spreading is measured in centimeters. A volume of 200 µL of the formulation was placed in the center of the base of the extensometer. Subsequently, weights of 26, 28, 31, 36, and 46 g were sequentially applied to the formulation, and the extensibility was measured after 1 min at each weight. The extensibility was measured as the diameters of the spreading formulation [23]. The experimental data were analyzed and fitted to mathematical models by means of GraphPad Prism^®^ version 6.0 (GraphPad Software Inc., San Diego, CA, USA).

### 2.6. In Vitro Release Study

In vitro release studies were carried out to assess the drug amount available from the selected formulation. To this end, Franz diffusion cells were used featuring a diffusion area of 0.64 cm^2^ and a receptor chamber volume of 4.9 mL. A SpectraPor^®^ regenerated cellulose dialysis membrane with a molecular cut-off weight of 14,000 Da (Sigma-Aldrich, Madrid, Spain) was hydrated for 24 h in methanol–water (1:1) to remove any residual impurities, then rinsed with Milli-Q water and afterwards soaked in receptor fluid for 4 h to equilibrate it before being mounted in the Franz diffusion cell (Crown Glass Company Inc., Jersey City, NJ, USA). Transcutol^®^ P–PBS 7.4 (1:1, *v*/*v*) was used as the receptor medium that provided the sink conditions throughout the study based on the drug’s solubility in the medium [22]. The assay was performed at 32 ± 0.5 °C by a circulating water jacket and the stirring speed was set at 500 r.p.m. to keep the contents uniform throughout the experiment. A total of 50 μL of the formulation was added to the donor compartment, and samples of 200 µL were withdrawn at the timepoints 3.0 h, 6.0 h, 8.30 h, 23.00 h, 26.30 h, 30.30 h, 40.00 h, 45.00 h, and 51.00 h. The same volume was promptly replaced by fresh Transcutol^®^ P–PBS 7.4 (1:1, *v*/*v*) to keep the volume constant throughout the study. This experiment was carried out using 5 replicates for each formulation. The samples obtained were analyzed by HPLC-FLD (see Section 2.3).

### 2.7. Ex Vivo Permeation Study Using Human Skin

The ex vivo permeation studies are useful to evaluate the ability of the substances to penetrate the skin, providing insights into the diffusion characteristics of the drugs. The permeation study assay was conducted with ex vivo human skin, as the biological membrane, mounted on the Franz diffusion cells. The skin was clamped between the donor and receptor chambers. The receptor compartment was filled with Transcutol^®^ P–PBS 7.4 (1:1, *v*/*v*) keeping sink conditions while being biocompatible with the skin [22]. The available diffusion area was 0.64 cm^2^, and the receptor fluid was kept at 32 °C and stirred continuously at 500 r.p.m. Before applying the formulation, a sample of the receptor fluid was withdrawn as control and then, 50 µL of formulation was placed in the donor compartment and 200 µL of receptor fluid was collected at predetermined time intervals (1, 2, 3, 4, 5, 7.5, 18.5, 21.5, and 23.5 h) and replaced with an equivalent volume of fresh receptor fluid. The amount of drug in the samples obtained from this study was quantified according to the HPLC-FLD (see Section 2.3). The experiment included five replicates and the results were reported as mean ± SD.

The permeation profile was evaluated as the cumulative amount of BCT permeated (µg) versus time (h). Different biopharmaceutical parameters were estimated including the flux (J_ss_, µg/(h/cm^2^)), which was determined as the slope of the linear section of the permeation profile using linear regression analysis. Other parameters such as lag time (TL, h), permeability coefficient (K_p_, (cm/h)), partition coefficient between vehicle and tissue (P_1_, cm), and the diffusion coefficient (P_2_, h^−1^) were also estimated [24]. The theoretical concentration that would be achieved in plasma after the topical application of the formulation in human (C_ss_) was also predicted starting from the flux and taking into account BCT’s plasma clearance [25] and a theoretical surface application of 10 cm^2^.

After completing the permeation study, the amount of drug that remained in the skin was extracted as follows: the skin was removed from the Franz diffusion cells and washed with distilled water. The permeation area was punched and weighed. Then, the skin discs were immersed in 1 mL of Transcutol^®^ P which acted as the extraction solvent. The drug was extracted from the skin by means of a sonication procedure for 10 min using an ultrasonic bath. The supernatant of samples was filtered and quantified by HPLC-FLD (see Section 2.3) to determine the amount of retained BCT in the tissue (Q_ret_).

### 2.8. Efficacy Study: Imiquimod-Induced Psoriasis Model

#### 2.8.1. Animals and Study Protocol

The study protocol received the approval from the Ethics Committee of BIOTERIO-UAEM de la Universidad Autónoma del Estado de Morelos (ref. 2023003 and date of approval on 12 May 2023). An imiquimod (IMQ)-induced psoriasis model was carried out using BALB/c mice to simulate the psoriasis symptoms in vivo. The animals remained in polypropylene cages at ambient temperature (20–25 °C) in a humidity-controlled (55%) environment with a light/dark cycle of 12 h, and given food and water ad libitum.

After a period of adaptation, the animals were randomly selected and divided into three groups according to experimental groups: negative control, positive control, and treatment group (*n* = 5 for each group). The mice in the negative control group were untreated healthy animals (no formulation or IMQ was applied). The animals in the positive group and BCT-OS group had psoriasis induced with 5 mg/mL of IMQ topically applied to the back skin and right ear for 6 consecutive days once daily and then the positive group was treated with 50 µL of PBS whereas the BCT-OS group was treated with 50 µL of the formulation once daily for another 6 days. At the end of the study, animals were euthanized by cervical dislocation and biopsy samples were extracted for histological analysis.

#### 2.8.2. Evaluation of Thickness and Biomechanical Skin Properties

During the study, the ear thickness of the mice in each group was measured using a Pocket Thickness Gage 7309 (Mitutoyo Corp., Kawasaki, Japan). This parameter was measured at different timepoints: day 0 (baseline), 6 (maximum inflammation), and 13 (last day of the trial). Additionally, the thickness of dorsal skin was measured after euthanizing the animals.

Biomechanical properties such as transepidermal water loss (TEWL) and stratum corneum hydration (SCH) were determined using a Tewameter measurement DermaLab1 module (Cortex Technology, Aalborg, Denmark) and a Corneometer CM-825 (Courage & Khazaka Electronics GmbH, Cologne, Germany), respectively. These parameters were measured at different timepoints: day 0 (basal), 6 (maximum inflammation), 8, 11, and 13 of the experiment.

#### 2.8.3. Histological Analysis

The samples of skin from the ear and back of the mice were fixed in a 4% formaldehyde solution for 24 h at 2 °C and then washed with PBS 3 times, replacing it with fresh medium at one-hour intervals. Samples were dehydrated in ethanol solutions and subsequently cleared in xylene. Finally, samples were embedded in paraffin twice, cut in 5 μm sections, stained with hematoxylin and eosin and observed to evaluate the histomorphology using a light microscope Olympus BX41 equipped with an Olympus XC50 camera (Olympus Co., Tokyo, Japan).

### 2.9. Evaluation of Biopharmaceutical Parameters in Healthy and Psoriatic Skin

An ex vivo permeation study was conducted using mouse skin, both psoriatic-induced skin and healthy skin. For this purpose, skin samples from the positive and negative control of the efficacy study were used for this assay. Ex vivo permeation studies were performed using Franz diffusion cells following the methodology described in Section 2.7. At the end of the experiment the amount of drug retained in the skin was determined and biopharmaceutical parameters such as TL, K_p_, P_1_, and P_2_ were also calculated according to Section 2.7.

### 2.10. Tolerance Study

#### 2.10.1. Tolerance by Evaluation of Biomechanical Skin Properties

The tolerance of the formulation was evaluated using Blank-OS (formulation with no drug). The biomechanical skin parameters such as transepidermal water loss (TEWL) and corneal hydration (SCH) were evaluated using the DermaLab1 module (Cortex Technology, Aalborg, Denmark) and a Corneomter CM-825 (Courage & Khazaka Electronics GmbH, Cologne, Germany), respectively. Ten healthy volunteers in the age range of 25–40 years participated in this study with prior informed consent and approval from the ethics committee of the University of Barcelona (IRB00003099; approved on 20 March 2018). Volunteers were asked to refrain from using cosmetics for 6 h prior to the test. They had acclimatization periods of approximately 30 min. Then, 0.5 mL/cm^2^ of Blank-OS was applied to the forearm of each volunteer. TEWL and SCH were measured in basal state and after formulation application at 10, 15, 30, 60, 120, and 180 min.

#### 2.10.2. Tolerance Study in Mouse

The in vivo tolerance of BCT-OS was assessed using BALB/c mice in accordance with the Ethics Committee of BIOTERIO-UAEM de la Universidad Autónoma del Estado de Morelos (code 2023004 approved on 12 May 2023). A negative control (non-treated mouse), positive control (mouse treated with xylol), and mouse treated with BCT-OS were used in this experiment. A volume of 50 µL of the selected formulation or xylol was topically administered to the back skin previously shaved. After 2 h of exposition, the animals were euthanized by cervical dislocation, and samples of skin were removed for histological analysis following the method described in Section 2.8.3.

## 3. Results

### 3.1. Design and Preparation of Lipid-Based Topical Solutions of BCT 5 mg/mL

The formulations T1, T2, and T3 showed drug precipitation after one day of preparation, and thus they were discarded from the study. The formulations T4, T5, and T6 showed a homogenous appearance for 30 days at both 30 and 40 °C. However, under these same study conditions, parameters such as pH and drug content were more stable for T4, indicating that the qualitative and quantitative composition of this formulation has better drug compatibility compared to T5 and T6 (Table 2 and Table 3), and therefore, T4 was selected as the final formulation of BCT-OS.

The selected formulation of BCT-OS was prepared by dissolving BCT in 40% Transcutol^®^ P, 20% of Labrafac^®^ Lipophile WL 1349, 28.2% Lauroglycol^®^ 90, and 11.3 of Surfadone^®^ LP 100) using a sonication process for 30 min until the complete solubilization of the drug.

### 3.2. Characterization of the Formulations

#### 3.2.1. Rheological Properties

**Figure 2:**
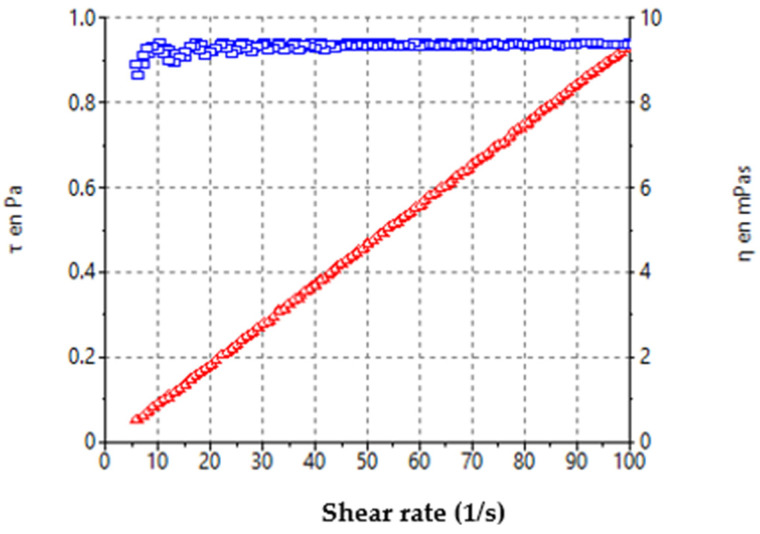
Rheological profile of BCT-OS. The viscosity curve is represented in blue color and the flow curve in red color.

The rheological behavior of the BCT-OS formulation is shown in Figure 2. BCT-OS exhibited a typical Newtonian profile, in which the relationship between the shear stress and the shear rate r was linear with an absence of thixotropy. The viscosity remained constant with a value of 9.38 ± 0.01 mPa·s at 100 s^−1^ at 25 ± 0.1 °C.

#### 3.2.2. Extensibility Assay

**Figure 3:**
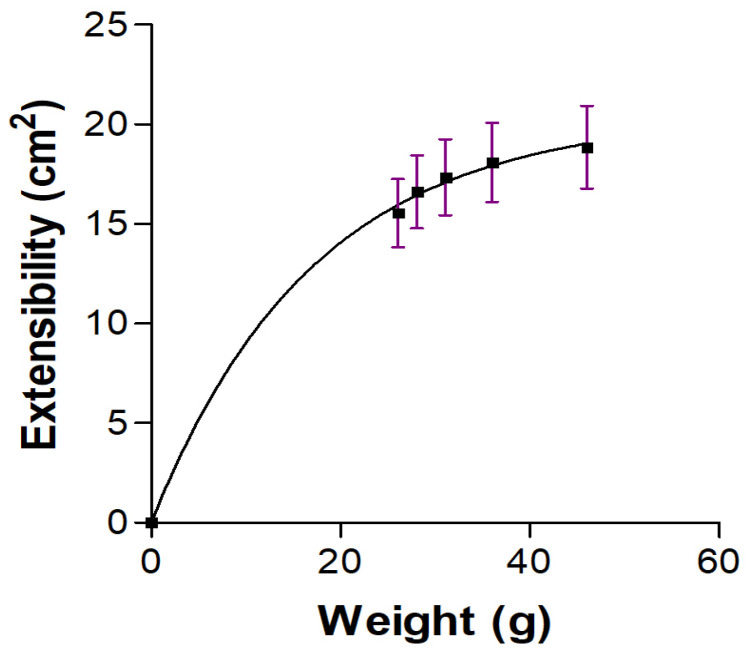
Extensibility profile of BCT-OS. Data are shown as the mean ± SD of three independent experiments (*n* = 3).

The extensibility assay involves subjecting the topical product to increasing weights to assess its ability to spread under pressure. This helps determine how the product behaves when applied with varying degrees of force, which can be important for user experience and product performance. The extensibility profile of BCT-OS followed a hyperbolic mathematical model with an r^2^ value of 0.9445 (Figure 3). Its low viscosity favored the easy spreadability of the formulation as the different weights were added until reaching a maximum extensibility of about 20 cm^2^.

### 3.3. In Vitro Release Study

**Figure 4:**
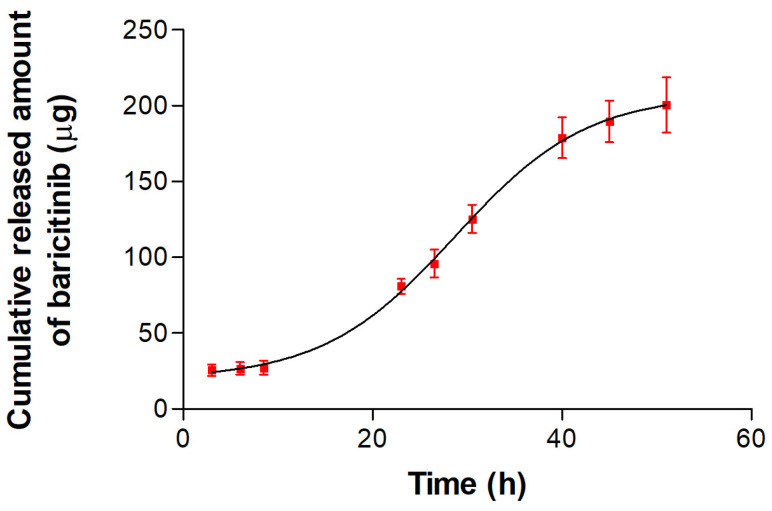
In vitro drug release profile of BCT from formulation. Data are presented as the mean ± SD (*n* = 5).

The drug release profile from the BCT-OS formulation is represented by the cumulative amounts of BCT released as a function of time, as shown in Figure 4. After 51 h of experiment, about 80% of the initial drug was released from the formulation.

### 3.4. Ex Vivo Permeation Study Using Human Skin

**Figure 5:**
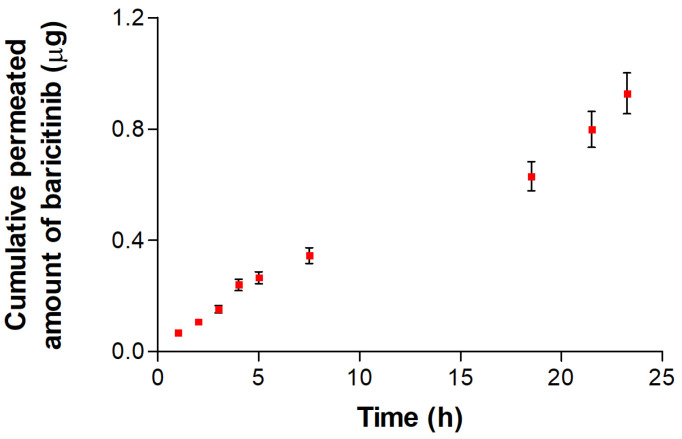
Permeation profile of BCT from the BCT-OS formulation through ex vivo human skin. Results are depicted as mean ± SD (*n* = 5).

Figure 5 shows the results of BCT permeation through human skin obtained from ex vivo studies. Only a minimal amount of drug was found in the samples extracted from the receptor fluid during the 23.5 h of study, indicating the difficulty of the drug in penetrating the deep layers of the skin.

Table 4 exhibits the permeation parameters calculated from the ex vivo permeation profile of BCT-OS. Results showed a drug flux (J_ss_) of 0.10 ± 0.02 µg/(h/cm^2^), a permeability coefficient (K_p_) of 0.19 ± 0.03 × 10^−4^ cm/h, vehicle/tissue partition coefficient (P_1_) of 0.14 ± 0.02 × 10^−4^ cm, and a diffusion coefficient (P_2_) of 1.40 ± 0.13 h^−1^. Additionally, the time taken for BCT to appear in the receptor fluid at a steady rate (Tl) was 8.42 ± 0.78 h. The theoretical steady-state plasma concentration (C_ss_) predicted was 0.06 ± 0.01 ng/mL, and the amount of BCT retained in the skin was 277.62 ± 52.75 µg/g skin/cm^2^.

### 3.5. Efficacy Study: Imiquimod-Induced Psoriasis Model

**Figure 6:**
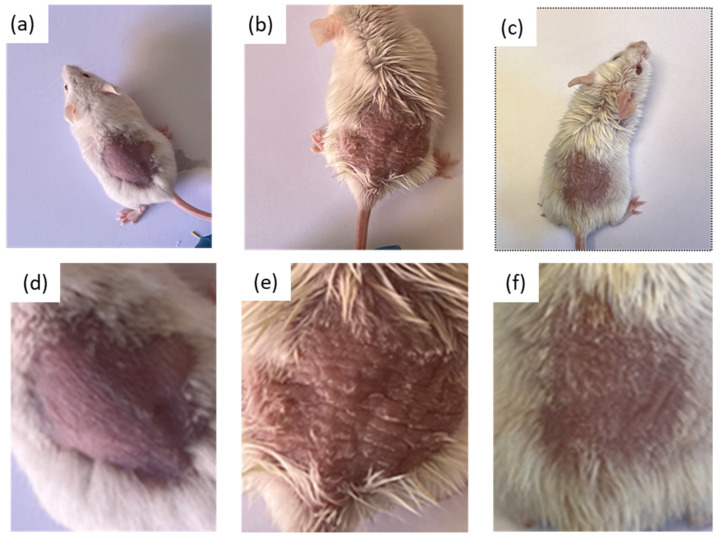
Photographs of the dorsal skin of mice on the last day of the experiment: negative control group (**a**,**d**); positive control group (**b**,**e**); and group treated with BCT-OS (**c**,**f**).

The topical exposure of IMQ to the skin of mice for 6 days induced the development of scaly lesions, erythema, and edema, characteristic symptoms of psoriasis (Figure 6b,e). However, daily treatment for 6 days with the formulation decreased these symptoms and progressively improved the appearance of the skin (Figure 6c,f).

#### 3.5.1. Evaluation of Thickness and Biomechanical Skin Properties

**Figure 7:**
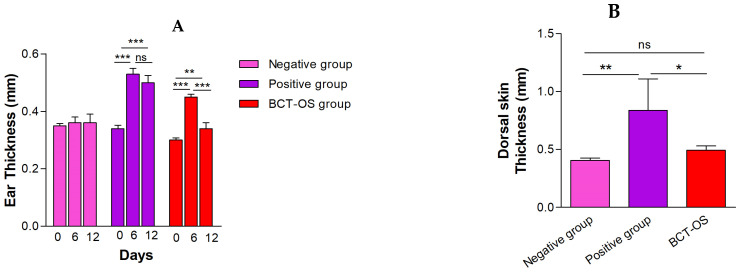
Evaluation of the skin thickness in (**A**) ear and (**B**) dorsal skin. Imiquimod was used to induce psoriasis in the positive group and in the BCT-OS group. Results are displayed as mean ± SD (*n* = 5). Significant statistical differences: * *p* < 0.05, ** *p* < 0.01, *** = *p* < 0.001, and ns, not significant.

After inducing inflammation for 6 days with IMQ, a significant increase in the thickness of mice ears was observed compared to the baseline (day 0). However, topical treatment with BCT-OS for 6 days after inducing psoriasis significantly reduced this parameter, alleviating the skin edema (Figure 7A). The thickness evaluation of the dorsal skin excised after sacrificing the experimental animals also confirmed these results: the positive control exhibited a markedly higher thickness compared to the BCT-OS treated group, which showed a similar value to the negative control group (Figure 7B).

**Figure 8:**
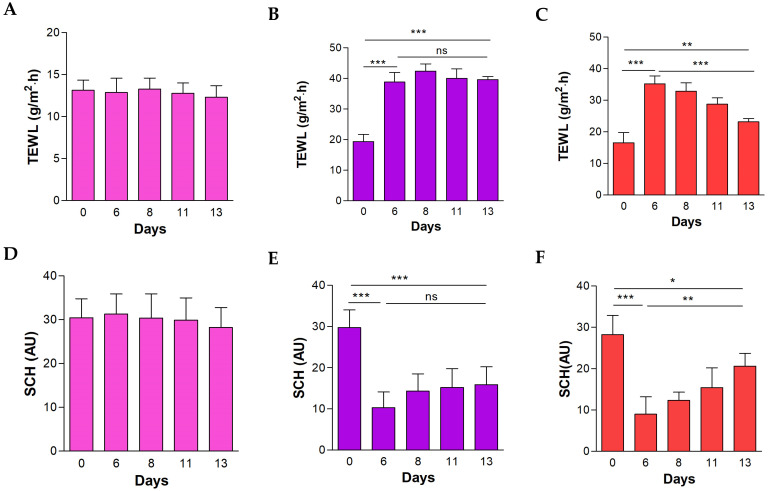
Evaluation of biomechanical skin properties in each experimental group using the imiquimod (IMQ)-induced psoriasis model. (**A**) Transepidermal water loss (TEWL) in the negative control group; (**B**) TEWL in the positive control group; (**C**) TEWL in the BCT-OS group; (**D**) stratum corneum hydration (SCH) in the negative control group; (**E**) SCH in the positive control group; and (**F**) SCH in the BCT-OS group. Results are expressed as mean ± SD (*n* = 5). Significant statistical differences: * *p* < 0.05, ** *p* < 0.01, *** = *p* < 0.001, and ns, not significant.

Applying IMQ topically to the skin notably raised the TEWL value while decreasing the SCH value, indicating damage to the skin barrier’s integrity, which aligns with the dryness and scaly lesions observed macroscopically on the dorsal skin of the mice after the sixth day of the experiment (Figure 8). Despite this, the group treated topically with the formulation significantly reduced the TEWL value and increased the SCH levels, showing a restoration of the biomechanical properties of the skin (Figure 8C,F).

#### 3.5.2. Histological Analysis

**Figure 9:**
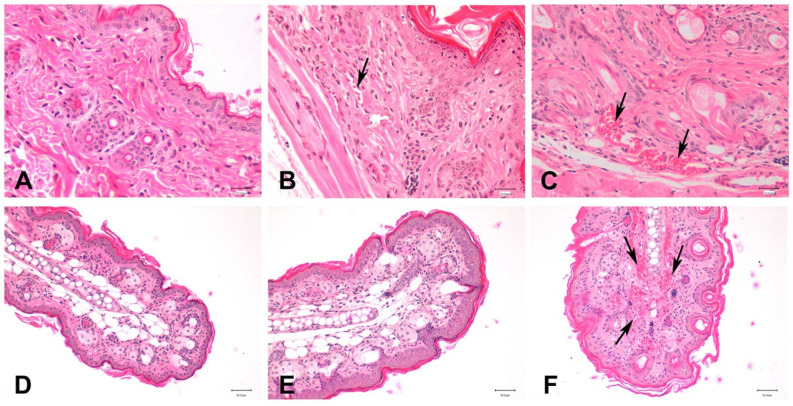
Histological images of ear and dorsal skin stained with hematoxylin and eosin. (**A**) Dorsal skin of the negative control group; (**B**) dorsal skin of the BCT-OS group; (**C**) dorsal skin of the positive control group; (**D**) ear skin of the negative control group; (**E**) ear skin of the BCT-OS group; and (**F**) ear skin of positive control group. Note the presence of dilated blood vessels (arrows) in the positive control, which are not observed or very diminished in the negative controls and treated tissues. Scale bar: (**A**–**C**) 25 µm; (**D**–**F**) 50 µm.

As shown in Figure 9A,D, healthy ear and dorsal skin in the negative control group exhibited an epidermis with normal appearance and an intact stratum corneum. The positive controls for ears and dorsal skin (Figure 9C,F) showed the presence of dilated blood vessels indicating an active inflammatory process induced by IMQ. The treatment with BCT-OS (Figure 9B,E) reduced the inflammation caused by IMQ showing a histological structure similar to the negative control with absent or very diminished dilated blood vessels.

### 3.6. Evaluation of Biopharmaceutical Parameters in Healthy and Psoriatic Skin

**Figure 10:**
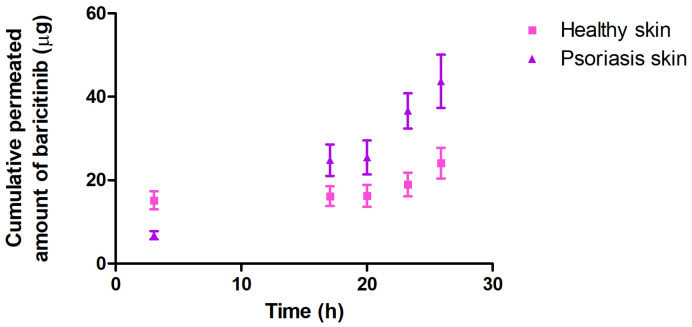
Permeation of BCT-OS through healthy and psoriatic ex vivo mouse skin. Results are expressed as mean ± SD (*n* = 5).

Figure 10 depicts the amount of BCT permeated through psoriatic and healthy mouse skin after 25 h of assay. A higher permeation of BCT was observed through mouse skin affected by psoriasis (43.68 µg) than through healthy mouse skin (24.10 µg).

The biopharmaceutical analyses described in Table 5 revealed that the values of J_ss_ and Tl were considerably and statistically significantly higher for the ex vivo studies on psoriatic mouse skin compared to those performed on healthy skin. On the other hand, Q_ret_ showed no significant differences.

### 3.7. Tolerance Study

#### 3.7.1. Tolerance Study by Evaluation of Biomechanical Skin Properties

**Figure 11:**
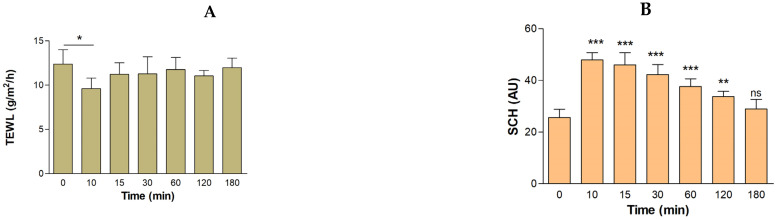
Assessment of the tolerance of the formulation in human individuals. (**A**) TEWL: transepidermal water loss and (**B**) SCH: stratum corneum hydration. Results are plotted as the mean and SD (*n* = 10). Statistically significant differences: * *p* < 0.05, ** *p* < 0.01, *** = *p* < 0.001, and ns, not significant.

Figure 11A illustrates a statistically significant decrease in the TEWL value after 10 min of application of the blank formulation to the skin. However, after 15 min, this parameter increased, progressively returning to the basal state. Regarding SCH, a significant increase was observed with respect to the basal state after 10, 15, 30, 60, and 120 min of topical treatment with the blank formulation. Nevertheless, a trend towards returning to the baseline state was noted after 3 h of the experiment (Figure 11B). The volunteers experienced no itching or pain during the evaluation of the formulation.

#### 3.7.2. Tolerance Study in Mouse

The tolerability of the formulation T4 was also evaluated in mice and compared to the negative control group, whose animals had been untreated, and to the positive control group, whose animals had been treated with xylol to cause inflammation.

**Figure 12:**
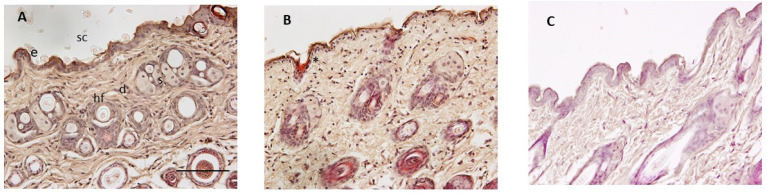
Representative histological sections of the mice skin in the tolerance study (original magnification, ×200). (**A**) Mouse skin of the negative control group; (**B**) group treated with xylol; and (**C**) group treated with BCT-OS. Skin structures: sc: stratum corneum e: viable epidermis; d: dermis; s: sebaceous gland; hf: hair follicle; and asterisk: loss of stratum corneum and epidermis. Scale bar = 100 µm.

Histologically, the negative control (Figure 12A) showed normal skin with a relatively thin epidermis and contiguous stratum corneum (SC). The topical application of xylol caused a loss of SC and epidermis. Finally, the skin treated topically with BCT-OS exhibited a similar pattern to that of the negative control group, with no signs of inflammation detected.

## 4. Discussion

In this study, the incorporation of BCT into an oily solution was used as a strategy to locally treat the inflammation and characteristic symptoms of psoriasis. BCT-OS was formulated using pharmaceutical and cosmetic components suitable for dermal administration. The correct selection of excipients is important to provide substantial drug incorporation, along with the formulation’s chemical and physical stability. Transcutol^®^ P, an ethylene oxide derivative, was used as a solvent due to its high solubilizing capacity for the drug as well as due to its high biocompatibility with the skin [7]. This excipient has been extensively used in pharmaceutics and cosmetics for the preparation of creams, emulsions, gels, ointments, and topical solutions, encompassing a broad spectrum of uses, including analgesics, anti-inflammatories, antifungals, hormones, and veterinary products [26]. Labrafac^®^ Lipophile WL 1349 and Lauroglycol^®^ 90 were used as secondary oily vehicles since both have been previously used in the development of formulations for the treatment of psoriasis [19]. Finally, Surfadone^®^ LP 100 was used as a penetration promotor to ensure the drug permeation through the stratum corneum [22].

BCT-OS exhibited appropriate organoleptic characteristics for topical application including excellent homogeneity and consistency, as well as a pH value of 5.60 which is biocompatible with the normal pH of the skin, guaranteeing non-irritating effects due to this parameter. Rheological properties of a topical formulation can modulate some biopharmaceutical parameters such as the release rate of the drug from its vehicle, as well as extensive coverage in the affected areas [27]. The rheological analysis confirmed the Newtonian behavior of BCT-OS at 25 °C (Figure 2), as anticipated in this type of formulation where there is a mixture of oils that create fairly fluid medications with low viscosity values. These low viscosity values facilitate packaging and administration by roll-on applications, or better yet, aerosolized to avoid touching the affected area [28]. The formulation showed high spreadability (Figure 3). The extensibility profile provides a measure of the deformation threshold of the vehicle. A topical formulation must exhibit high extensibility, since this means that it is not necessary to apply too much pressure to spread the product on the area to be treated [29,30,31,32,33] and considering that the product would be applied on inflamed psoriatic areas, it is ideal that its administration is easy and comfortable for the patient. The developed formulation is highly fluid and extensible, making it ideal for spray application. It can be easily applied to large areas of the body, such as the legs, abdomen, or arms, where psoriasis may occur.

The in vitro release study is relevant for evaluating the performance of topical products, to ensure that the drug is delivered effectively, since the drug release affects the permeation rate of the drug and therefore defines its bioavailability [34]. It is necessary to confirm that the drug is able to be released from the formulation before continuing with the skin permeation studies, since in the case that the drug does not permeate, we could to be able to elucidate the reason, whether it is due to the inability of the drug to be released from the vehicle or the inability to permeate. In the event that the drug was not easily released, it would be necessary to make appropriate adjustments and reformulations. The results for BCT-OS showed that approximately 200 µg of BCT were released from the oily vehicle, which represents about 80% of the initial dose of the drug (Figure 4). This result indicates that this formulation is capable of releasing the drug, and therefore it will not hinder the drug’s permeation through the skin. In other words, the drug release will not be a rate-limiting step in the drug’s permeation through the skin.

The success of locally acting dermal treatments depends on the ability of the drug to penetrate the stratum corneum, diffuse through the deeper layers of the skin, and remain within the target area for the time necessary to exert its action [35,36]. Ex vivo permeation studies revealed that BCT persists in the skin and only a minimal amount of drug reached the receptor compartment (Figure 5), thereby demonstrating that BCT-OS could serve as a local cutaneous therapeutic approach without significant adverse systemic effects. Regarding the biopharmaceutical analysis (Table 4), the predominance of P_2_ over P_1_ facilitates the drug’s diffusion through the epidermis and dermis. The high Tl value of 8.42 h means that during this time, the drug is diffusing through the skin and being distributed into it, ensuring a prolonged local effect and minimizing systemic adverse reactions. Previous studies have reported maximum plasma concentrations of 24.6 ng/mL after the oral administration of 2 mg BCT tablets [37]. Therefore, the low C_ss_ value of 0.06 ng/mL obtained in this study allows the avoidance of the systemic immunosuppressive effect of BCT, thus ensuring only a local anti-inflammatory activity. The high amount of BCT retained inside the skin (277.62 µg/g skin/cm^2^) demonstrated that the drug is able to cross the stratum corneum and remain within the deeper layers of the skin, facilitating a local and prolonged effect of the drug. This result indicates that the oily solution enhances the retention of the drug in the target area, promoting its duration of action, which could consequently reduce the frequency of administration in patients.

In the IMQ-induced psoriasis model study, the results showed an increase in the thickness of the ear and dorsal skin of the animals as a result of the edema caused by the topical exposition to IMQ, but these thickness values decrease significantly with BCT-OS treatment. This effect could be explained by the anti-inflammatory activity already reported for BCT [38]. Biomechanical properties of the skin including TEWL and SCH were also evaluated. Specifically, in the negative control group (healthy mice), the TEWL values remained constant for the 13 days of the study. However, the topical application of IMQ on the skin caused an increase in these values of up to double compared to the basal state. Likewise, the SCH values decreased to approximately half of baseline values at the end of the experiment. This increase in TEWL and decrease in SCH is a consequence of the damage to the skin integrity caused by the topical exposure to IMQ, generating itching, redness, inflammation, and dryness, which was observed macroscopically on the affected skin (Figure 6) [39]. However, after applying the treatment with BCT-OS, the TEWL and SCH values were reestablished, which could be explained by the mixture of oils contained in the formulation, since oily substances help retain moisture and reduce the TEWL, improving skin hydration [40]. In addition, the application of oily formulations softens the surface of the skin by occupying the spaces between partially peeling skin scales (as in the case of psoriasis) and restores the capacity of intercellular lipid bilayers to absorb, retain, and redistribute water [41].

Regarding the histology in the positive control (Figure 9C,F), a dilation of the blood vessels can be observed, which is not observed in the ear treated with BST-OS (Figure 9E), and a decrease in these vessels can be seen on the back with the treatment applied (Figure 9E). These dilated blood vessels usually occur in inflammatory processes and have been reported in skin psoriasis, which manifests itself in the red color of the lesions as observed in this study [42,43,44]. Based on these encouraging findings, subsequent biochemical studies will be necessary and complementary to test the effectiveness of this formulation in animal models with the disease.

The ex vivo permeation study using healthy and psoriatic mouse skin showed that drug permeation and flux in psoriatic skin were about twice as high (43.68 µg and 3.100 µg/h, respectively) as those obtained in healthy skin (24.10 µg and 1.310 µg/h, respectively), expected results considering that the integrity of the skin is compromised in psoriasis, making it more susceptible to the transfer of substances across the skin barrier (Figure 10 and Table 5).

The tolerability of BCT-OS was studied by assessing the biomechanical properties of the skin, including TEWL and SCH, parameters widely used to evaluate the integrity of the skin barrier after being exposed to physical or chemical agents [45]. The results suggest that the vehicle is a safe formulation, as they showed a decrease in the TEWL values (more marked at 10 min) and an increase in SCH values after the application of the blank oily solution. These results could be due to the occlusive effect generated by the formulation which creates a hydrophobic barrier that blocks transepidermal water loss [20]. The mixture of oils could be covering small cracks in the skin providing a moisturizing effect, and a soothing and protective film. However, these TEWL and SCH values tended to revert to their baseline levels, concluding that there are no significant changes in terms of biomechanical parameters, and thus BCT-OS does not cause structural changes in the skin. This conclusion was supported by the tolerance study in mice, where histopathological analysis revealed no infiltration of inflammatory cells or notable changes in the skin compared to the negative control after the topical application of BCT-OS, which suggests that this formulation could be non-irritating and gentle on the skin.

## 5. Conclusions

Based on the findings of the research, the lipid-based formulation of baricitinib (BCT-OS) has demonstrated promising results in the topical treatment of psoriasis. This formulation showed controlled release and convenient skin permeation parameters, which are critical to ensure the drug reaches the desired target within the skin layers. Additionally, the BCT-OS formulation exhibited significant anti-inflammatory efficacy in an imiquimod-induced psoriasis model in mouse, suggesting its potential to alleviate symptoms of psoriasis. The formulation was well tolerated, with no adverse effects on skin integrity, highlighting its safety for prolonged use. These findings contribute significantly to the development of new alternatives to treatments for psoriasis. By offering localized therapeutic benefits and minimizing systemic side effects typically associated with oral administration, the BCT-OS formulation represents a potential breakthrough in psoriasis treatment, particularly for patients who experience adverse effects from systemic therapies.

## Figures and Tables

**Table 1:** Composition of the lipid-based formulations with different oily content. Quantities are expressed as percentages.

Formulations	Baricitinib (%)	Transcutol^®^ P (%)	Labrafac^®^-Lipophile WL 1349 (%)	Lauroglycol^®^ 90/Surfadone^®^ LP 100 (5:2) (%)
T1	0.5	20	20	59.5
T2	0.5	20	40	39.5
T3	0.5	20	60	19.5
T4	0.5	40	20	39.5
T5	0.5	40	40	19.5
T6	0.5	60	20	19.5

**Table 2:** Physical and chemical characterization of lipid-based topical solutions of BCT 5 mg/mL after one day of preparation and reevaluation at thirty days of preparation stored at 30 ± 2 °C/65 ± 5% RH.

Formulations	One Day		Thirty Days	
	pH	Drug Content (%)	pH	Drug Content (%)
T4	5.60 ± 0.12	99.02 ± 0.10	5.47 ± 0.09	98.95 ± 0.12
T5	5.57 ± 0.10	98.97 ± 0.18	5.06 ± 0.13	98.01 ± 0.13
T6	5.45 ± 0.15	99.08 ± 0.09	4.73 ± 0.07	98.16 ± 0.15

**Table 3:** Physical and chemical characterization of lipid-based topical solutions of BCT 5 mg/mL after one day of preparation and reevaluation at thirty days of preparation stored at 40 ± 2 °C/75 ± 5% RH.

Formulations	One Day		Thirty Days	
	pH	Drug Content (%)	pH	Drug Content (%)
T4	5.60 ± 0.12	99.02 ± 0.10	5.38 ± 0.07	98.87 ± 0.10
T5	5.57 ± 0.10	98.97 ± 0.18	4.92 ± 0.12	97.92 ± 0.16
T6	5.45 ± 0.15	99.08 ± 0.09	4.35 ± 0.15	97.89 ± 0.12

**Table 4:** Permeation parameters of BCT-OS through ex vivo human skin. Results are reported as mean ± SD (*n* = 5).

Parameters	Mean ± SD
J_ss_ (µg/(h/cm^2^))	0.10 ± 0.02
K_p_ (×10^−4^ cm/h)	0.19 ± 0.03
Tl (h)	8.42 ± 0.78
P_2_ (h^−1^)	1.40 ± 0.13
P_1_ (×10^−4^ cm)	0.14 ± 0.02
C_ss_ (ng/mL)	0.06 ± 0.01
Q_ret_ (µg/g skin/cm^2^)	277.62 ± 52.75

Abbreviations: J_ss_ (flux), K_p_ (permeability coefficient), Tl (lag time), P_1_ (vehicle/tissue partition coefficient), P_2_ (diffusion coefficient), C_ss_ (steady-state plasma concentration), and Q_ret_ (amount of drug retained in the tissue).

**Table 5:** Permeation parameters of BCT-OS through healthy and psoriasis mouse skin. Results are reported as mean ± SD (*n* = 5).

Parameters	Healthy Skin	Psoriasis Skin	Statistical Significance
J_ss_ (µg/h)	1.310 ± 0.138	3.100 ± 0.093	***
Tl (h)	7.89 ± 0.680	11.68 ± 1.250	***
Q_ret_ (µg/mg skin)	0.5320 ± 0.064	0.4880 ± 0.570	ns

Abbreviations: J_ss_ (flux), Tl (lag time), and Q_ret_ (amount of drug retained in the skin). *** = *p* < 0.001, and ns = not significant.

## Data Availability

The data presented in this study are available in this article.
